# Supplementary material for: Nonlinear Changes in Botulinum Toxin Treatment of Task-Specific Dystonia during Long-Term Treatment
Source: Toxins (Basel). 2021 May 22;13(6):371. doi: 10.3390/toxins13060371 (PMC8224565; doi:10.3390/toxins13060371)
Supplement: Supplementary file 1 [file toxins-13-00371-s001.zip › toxins-1179596-SI.pdf]

Nonlinear Changes in Botulinum Toxin Treatment of Task-Specific Dystonia during Long-Term Treatment

André Lee, Jabreel Al-Sarea and Eckart Altenmüller

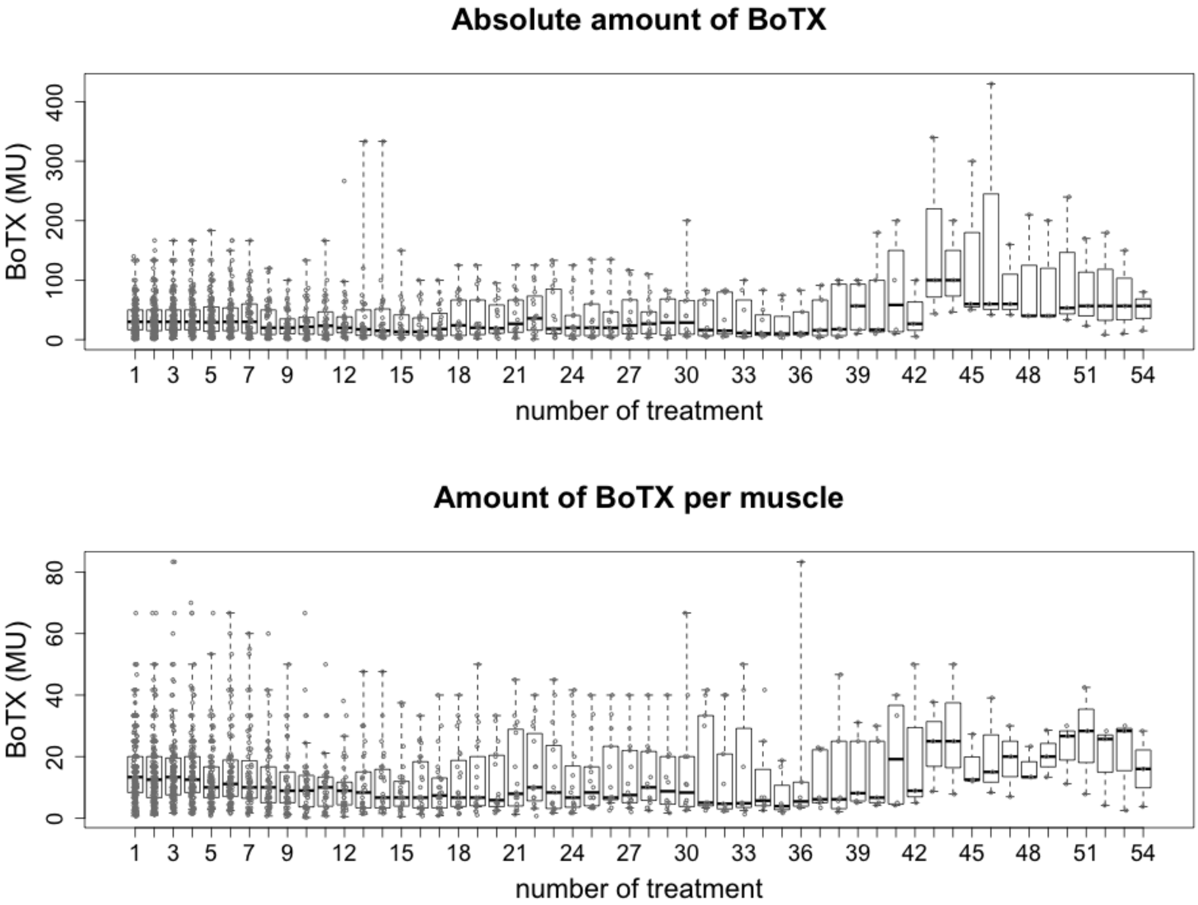

Figure S1. Boxplots of the absolute amount of BoTX (top) and the relative amount of BoTX (bottom) per treatment. Each boxplot represents one treatment session with the small grey circles depicting individual patients' data.

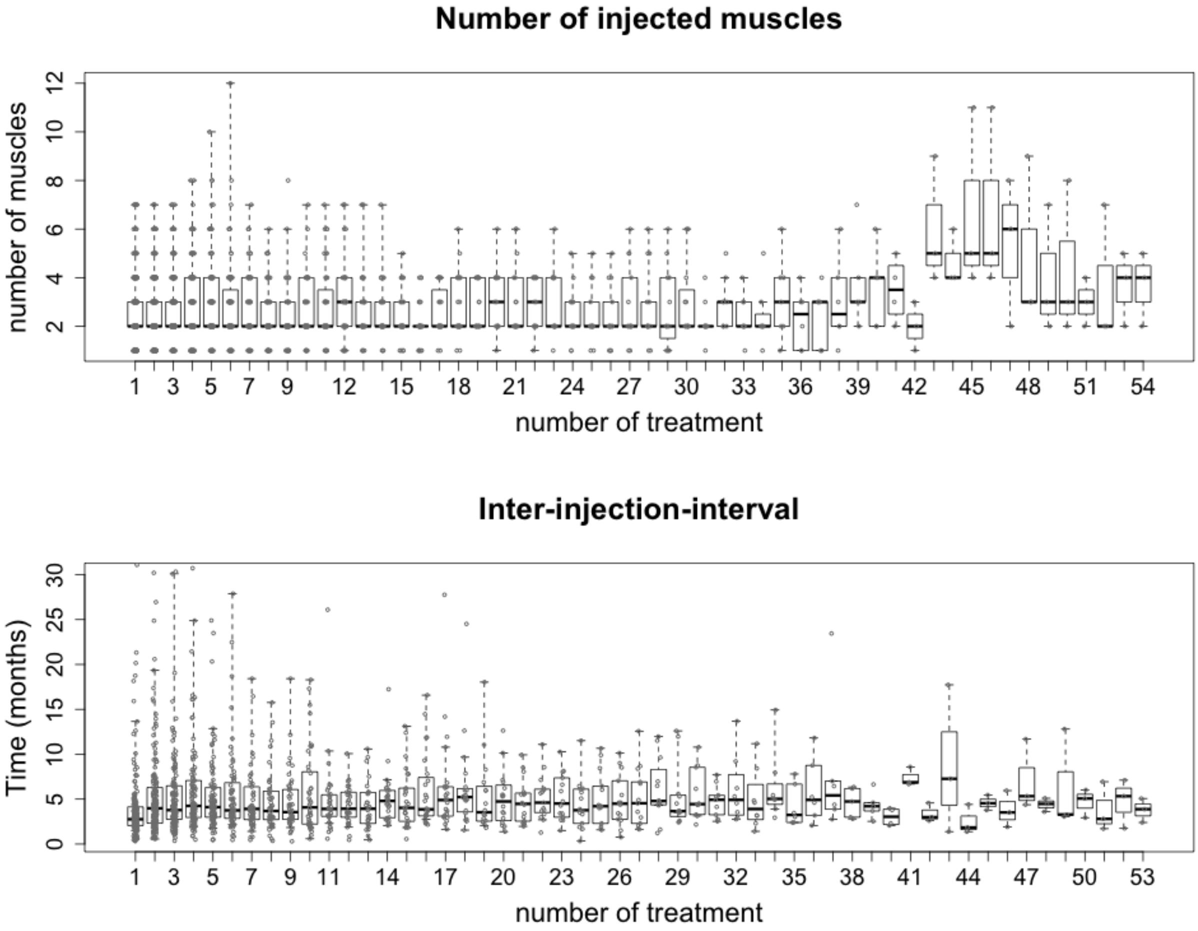

Figure S2. Boxplots of the number of injected muscles (top) and the inter-injection interval (bottom) per treatment. Each boxplot represents one treatment session with the small grey circles depicting individual patients' data.
